# Mexico's path towards the Sustainable Development Goal for health: an assessment of the feasibility of reducing premature mortality by 40% by 2030

**DOI:** 10.1016/S2214-109X(16)30181-4

**Published:** 2016-08-30

**Authors:** Eduardo González-Pier, Mariana Barraza-Lloréns, Naomi Beyeler, Dean Jamison, Felicia Knaul, Rafael Lozano, Gavin Yamey, Jaime Sepúlveda

**Affiliations:** a Mexican Health Foundation, Mexico City, Mexico; b Independent consultant, Palo Alto, CA, USA; c Global Health Sciences, University of California, San Francisco, San Francisco, CA, USA; d Global Health Sciences, University of California, San Francisco, San Francisco, CA, USA; e Miami Institute for the Americas, University of Miami, Coral Gables, FL, USA; f Mexican Health Foundation, Mexico City, Mexico; g Center for Health Systems Research, National Institute of Public Health, Cuernavaca, Mexico; h Duke Global Health Institute, Duke University, Durham, NC, USA; i National Institute of Public Health, Cuernavaca, Mexico

## Abstract

**Background:**

The United Nations Sustainable Development Goal for health (SDG3) poses complex challenges for signatory countries that will require clear roadmaps to set priorities over the next 15 years. Building upon the work of the Commission on Investing in Health and published estimates of feasible global mortality SDG3 targets, we analysed Mexico's mortality to assess the feasibility of reducing premature (0–69 years) mortality and propose a path to meet SDG3.

**Methods:**

We developed a baseline scenario applying 2010 age-specific and cause-specific mortality rates from the Mexican National Institute of Statistics and Geography (INEGI) to the 2030 UN Population Division (UNPD) population projections. In a second scenario, INEGI age-specific and cause-specific trends in death rates from 2000 to 2014 were projected to 2030 and adjusted to match the UNPD 2030 mortality projections. A third scenario assumed a 40% reduction in premature deaths across all ages and causes. By comparing these scenarios we quantified shortfalls in mortality reductions by age group and cause, and forecasted life expectancy pathways for Mexico to converge to better performing countries.

**Findings:**

UNPD-projected death rates yield a 25·9% reduction of premature mortality for Mexico. Accelerated reductions in adult mortality are necessary to reach a 40% reduction by 2030. Mortality declines aggregated across all age groups mask uneven gains across health disorders. Injuries, particularly road traffic accidents and homicides, are the main health challenge for young adults (aged 20–49 years) whereas unabated diabetes mortality is the single most important health concern for older adults (aged 50–69 years).

**Interpretation:**

Urgent action is now required to control non-communicable diseases and reduce fatal injuries in Mexico, making a 40% reduction in premature mortality by 2030 feasible and putting Mexico back on a track of substantial life expectancy convergence with better performing countries. Our study provides a roadmap for setting national health priorities. Further analysis of the equity implications of following the suggested pathway remains a subject of future research.

**Funding:**

Mexico's Ministry of Health, University of California, San Francisco, and Bill & Melinda Gates Foundation.

## Introduction

The Sustainable Development Goal for health (SDG3), adopted in September 2015, poses enormous challenges for all countries. Underpinning the overall objective to ensure healthy lives and promote wellbeing for all at all ages is a list of nine proposed targets (appendix).^1^ Previous analyses undertaken to inform the SDG3 targets provide evidence and proposals on target measurement and on what could be achievable at a global level based on observed country-specific mortality.^2^, ^3^, ^4^ These analyses show that country roadmaps to meet the SDG3 targets require a deeper analysis of specific mortality trends and a pragmatic approach to linking targets with cost-effective health interventions.^3^, ^5^

Norheim and colleagues^3^ proposed an overarching quantitative target to support SDG3, namely to avoid 40% of premature deaths by 2030 (premature deaths defined as those of individuals younger than 70 years), which in turn would be the result of achieving a series of cause-specific mortality subtargets (appendix).^3^ This ambitious undertaking can be reached only by clearly understanding the main causes of death by age group and the effective interventions available to affect those causes and their respective modifiable risk factors. Countries have the opportunity to apply specific and unique policy strategies targeted to their national health priorities and health system capabilities. Therefore, there are different ways to achieve a so-called 40 by 30 (40 × 30) overall target.

The *Lancet* Commission on Investing in Health^6^ showed that a grand convergence in health (a reduction in avertable infectious, child, and maternal mortality down to universally low levels) could be achieved by 2035. Since publication of its report titled Global Health 2035, the Commission on Investing in Health working group has embarked on a series of consultations with donor agencies and ministries of health and finance to explore the implications for investments in health. One of these engagements has been with Mexico's Ministry of Health on how to achieve SDG3 and get on track to converge to the better performing high-income countries (eg, among the Organization for Economic Cooperation and Development [OECD] countries of which Mexico is a member country).^7^ Mexico achieved major improvements in preventable maternal, newborn, and child health (MNCH), and substantial gains in life expectancy over the past 30 years. Despite this, Mexico is still behind most other OECD countries in several key areas ranging from comparatively high mortality from MNCH causes to the rising rates of non-communicable diseases (NCDs) and fatal injuries. As a result, life expectancy increase has slowed and is far from convergence with OECD countries (panel 1).^8^

**Research in context**

**Evidence before this study**

Our work builds on the *Lancet* Commission on Investing in Health and Norheim and colleagues' work. These sources provide a robust general framework for global convergence to explore how Mexico can reach Sustainable Development Goal 3 (SDG3). We build upon these analyses using United Nations Population Division age-specific mortality estimates and Mexican National Institute of Statistics and Geography age-specific and cause-specific mortality data, calibrating our results to the United Nations life expectancy projections for international comparability. We identified best-practice interventions based on the recommendations of international organisations and initiatives including WHO, the Organisation for Economic Co-operation and Development, and Disease Control Priorities, Third Edition.

**Added value of this study**

Our findings shed light on the underlying causes of mortality by age group that are limiting Mexico's potential to achieve SDG3 and thus, convergence in health status. Our study provides a country roadmap to identify and prioritise the health interventions that can support achievement of SDG3 and reduce the gap in life expectancy by 2030. Our approach can be used by other countries to operationalise SDG3.

**Implications of this study**

Our study can assist national authorities to prioritise areas of action with the greatest health impact and economic returns. Effective priority setting is important in the face of budgetary constraints, and in evidence-based dialogue with other governmental authorities, such as the Ministry of Finance, and with other non-health sectors whose actions have an effect on health (eg, transport, education, and violence prevention).

In this study we project shortfalls, relative to a target of a 40% reduction of premature deaths, in age-specific and cause-specific mortality in Mexico by comparing three different mortality scenarios in 2030. Following the Commission on Investing in Health^6^ notion of grand convergence, we use these results to forecast life expectancy at birth and analyse Mexico's potential to meet SDG3 and reach substantial convergence to better performing high-income countries.

## Methods

Annual population estimates for 1990–2014 and projections based on the medium fertility variant for 2015–30 were taken from the United Nations Population Division (UNPD) World Population Prospects 2015 revision. The UNPD 5-year periods for mortality estimates were averaged to obtain midpoint estimates; these in turn were averaged to obtain estimates for every fifth year between 1990 and 2030. Intermediate years were then constructed assuming linear trends between these 5-year estimates. Population and deaths were grouped into five age groups: 0–4 years, 5–19 years, 20–49 years, 50–69, and 70 years or more.

We used cause-specific mortality estimates from the vital statistics reported by the National Institute of Statistics and Geography (INEGI). The INEGI cause-specific mortality data were combined with UNPD mortality and population estimates and projections to construct three mortality scenarios. INEGI annual reported deaths between 1990 and 2014 were grouped into 15 major categories representative of the most important disease clusters to obtain annual distributions of deaths by category across each of the five age groups (appendix). Deaths with an ill-defined or otherwise unspecified cause (International Classification of Diseases, 10th Revision [ICD-10] codes R00–R99; 2·3% for 1990 and 1·7% for 2014) or unspecified age (0·7% for 1990 and 0·5% for 2014) were redistributed using the observed cause and age distributions, respectively. The resulting cause-specific distributions for the five age groups were applied to the UNPD deaths and population figures to produce adjusted figures for deaths and death rates by age group and disease category from 1990 to 2014.

We constructed three scenarios with age-specific and cause-specific mortality estimates for 2030. The baseline scenario reflects changes in mortality exclusively associated with population growth and aging, and does not account for changes in mortality rates. Mortality rates were assumed to remain at the 2010 levels. Therefore, the 2010 age-specific and cause-specific mortality rates were applied to the 2030 UNPD population prospects. We used 2010 as reference year to ensure consistency with the SDG3 and the work of Norheim and colleagues.^3^

The so-called inertial scenario projects cause-specific mortality for 2030 assuming recent (2000–14) trends will continue so that expected progress is limited to merely letting policies and mortality trends follow their present path. With the statistical software R, we produced mortality rate projections for 2015–30 by disease category for each age group within the 0–69 years age range using a linear regression model based on the INEGI–UNPD adjusted 2000–14 mortality rates. The projected mortality rates were applied to the UNPD population structure to produce estimates of deaths for 2015–30. These distributions across disease categories for all years and age groups were then adjusted to the UNPD 2015–30 medium-variant expected premature deaths for each age category to obtain cause-specific UNPD compatible estimates for deaths and death rates across the entire 1990–2030 time period.

**Figure 1:**
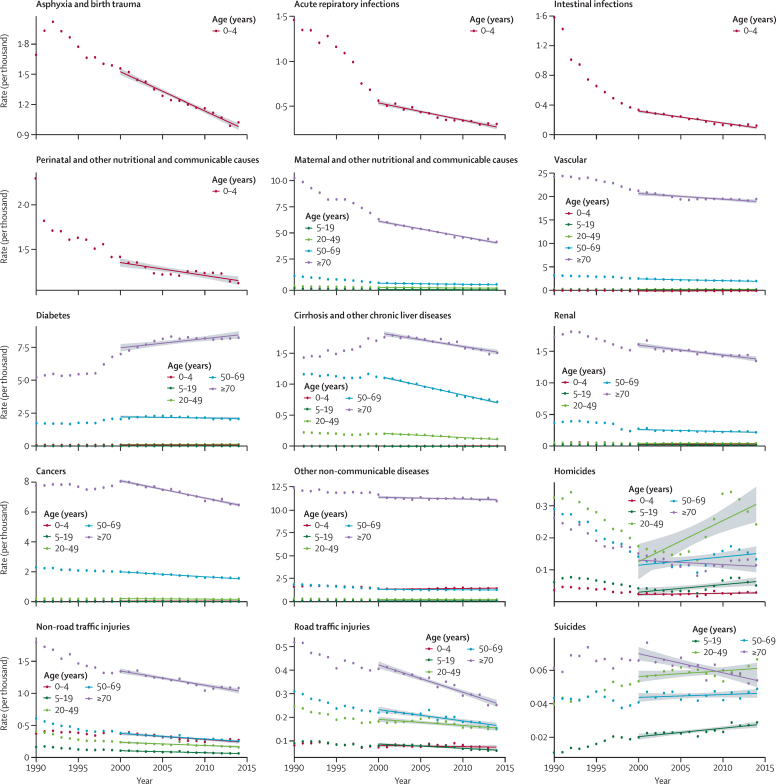
Mortality trends by major disease group, Mexico 1990–2014 Own estimates using combined data from the United Nations, Department of Economic and Social Affairs, Population Division, World Population Prospects, 2015 Revision, and the National Institute of Statistics and Geography, Statistics of mortality (INEGI; 1990–2014). Lines correspond to the 2000–2014 trends based on a simple linear regression. The shaded area around each fitted regression corresponds to the 95% CI. The list of ICD-9 and ICD-10 codes used to group deaths by major cause is included in the appendix.

We used trends for the 2000–14 period (instead of the 1990–2014 period) to show the more recent evolution of mortality and to avoid potential biases from coding in 1998, when the ICD-10 was introduced in Mexico. Linear trends show a particularly good fit for most causes for the 2000–14 period. In the case of homicides, however, the use of this timeframe leads to an overestimation of the expected number of deaths in this inertial scenario because of the large increase in the homicide rate between 2005 and 2011.^34^ Nevertheless, we used a single timeframe for purposes of consistency. The underlying trends used to estimate this scenario are shown in figure 1.

The SDG 40 × 30 scenario uses the baseline scenario as the departure point and projects the number of deaths and the resulting death rates consistent with a target of a 40% reduction in overall premature mortality by 2030. We assumed a flat 40% rate of reduction across each age group in the 0–69 years age range and for each of the 15 causes. We compared deaths and death rates between the SDG 40 × 30 estimated target and the inertial scenario to identify disease and age groups and resulting policy areas that are off-track and need scaling up of interventions to accelerate mortality reductions, and thus meet SDG3 targets. We calculated life expectancy based on the inertial and SDG 40 × 30 scenarios to assess convergence, with Japan as the best performer and the USA as an underperformer in the OECD. The USA is a particularly relevant reference for Mexico given its large population of Mexican origin and because it faces similar health challenges, most notably rising levels of overweight and obesity, as well as an ageing population.

In order to develop a complete profile of mortality in all age groups over time, we included results for the population aged 70 years and older. For this age group, the baseline and inertial scenarios follow the same logic as for the 0–69 years age group, except that the cause-specific analysis was not undertaken. Baseline figures reflect the demographic effect of an aging population by keeping the 2010 mortality rate constant. Figures for the inertial scenario reflect the number of deaths as projected by UNPD, whereas the SDG 40 × 30 scenario assumes the same mortality as the inertial scenario.

### Role of the funding source

This study was partly supported by the Ministry of Health in Mexico, the Bill & Melinda Gates Foundation, and the University of California, San Francisco. The funders of this study had no role in the study design, or conduct, data collection, data management, data, analysis, data interpretation, or writing of the report. The corresponding author, as Under-Secretary of Health of Mexico from 2014 to March, 2016, was responsible for the General Directorate of Health Information, which provides information on vital statistics in coordination with INEGI. The corresponding author had full access to all the data in the study and had final responsibility for the decision to submit for publication.

## Results

According to UNPD estimates, the Mexican population will age substantially in the coming decades and grow from 118·6 million people in 2010 to 148·1 million people in 2030. Under the baseline scenario, total mortality is projected to increase by 82% between 2010 and 2030 (table 1). Nearly half of this increased mortality (46%) is a product of population aging, which is projected to result in more than 810 000 additional deaths by 2030. The remaining almost 202 000 additional deaths are the result of population growth. Most of the increased mortality occurs in older populations. Premature mortality (at age 0–69 years) is projected to increase by 49% and mortality at age 70 years and older is projected to increase by 122%.

The SDG 40 × 30 scenario seeks to avert 40% of yearly premature deaths in 2030 compared with the baseline scenario. However, UNPD projections for 2030 show that premature mortality will fall by only 26%, which is almost 64 000 deaths short of the SDG 40 × 30 target. Although mortality rates decreased for all age groups between 1990 and 2010 and are expected to drop further by 2030, the overall rate of decrease has slowed substantially and masks varying trends in mortality across age groups. From 1990 to 2010 the death rate for children aged 0–4 years decreased by 48%, whereas for older adults (aged 50–69 years) it decreased by only 29%. From 2010 to 2030, reductions in mortality across all ages will be slower than in the previous two decades; however, mortality reductions in children and adolescents (aged 0–19 years) will continue to outpace gains in adults (aged 20–69 years). Under the inertial scenario, the decrease in premature mortality will be 42% for children aged 0–4 years and 33% for young people aged 5–19 years. By contrast, the decrease for adults aged 20–49 years will be 25% and for those aged 50–69 years it will be 23%.

The slower rates of decrease in adult mortality are of particular concern. Under the baseline scenario, 85% of premature deaths will occur in people aged 20–69 years, precisely where the least health gains are expected in the future. In fact, nearly all (98·5%) of the predicted shortfall in mortality reduction comparing the inertial with the SDG 40 × 30 target will be in those aged 20–69 years (about 17 000 excess deaths for those aged 20–49 years and 47 000 excess deaths for those aged 50–69 years).

Mortality trends by major cause of death further explain the mortality dynamics seen across age groups. Trends used to construct the inertial scenario are shown in figure 1. In the baseline scenario, 13% of total premature mortality is associated with MNCH disorders and communicable diseases (table 2). By contrast, 70% is associated with NCDs, and 17% with injuries (see combined results by cause and age group in the appendix).

Childhood infectious diseases and adverse events associated with childbirth continue to represent an unfinished health agenda. Nevertheless, mortality from communicable, perinatal, maternal, and nutritional causes shows the most impressive downward trends up to 2030. Under the inertial scenario, the SDG 40 × 30 targets for MNCH disorders, as well as mortality targets for children and adolescents (aged 0–19 years), are expected to be met by a good margin (table 1). However, sustained efforts to ensure that recent trends continue for this cluster will be necessary to attain the target.

A cluster of four major non-communicable disease groups, vascular disease (including cardiovascular diseases, stroke, and hypertensive diseases), diabetes, renal disease, and liver disease, will account for more than 40% of total premature deaths in the baseline scenario, maintaining their present position as a leading cause of premature mortality. From 1990 to 2010, the share of mortality from this cluster, relative to total premature deaths, increased from 72 714 (23·1%) to 103 573 (34·1%), and deaths from these causes were 1·4 times higher in 2010 than in 1990. Deaths from cancer also increased during this period, though their contribution to premature mortality increased less, from 28 640 (9·1%) to 37 669 (12·4%).

The largest challenge and the greatest opportunity to meet the SDG 40 × 30 target therefore lies in reversing the trends for mortality from NCDs. The inertial scenario will fall short of this goal by about 54 000 deaths in 2030. Half of this shortfall results from the very small projected reduction in diabetes mortality. Under present trends, expected mortality from diabetes will nearly double by 2030, and will be 1·6 times higher than the SDG 40 × 30 target. Similar challenges exist for renal disease and a diverse set of other NCDs (such as non-congenital thyroid and other endocrine disorders, mental and behavioural disorders, or other nervous, eye, ear, respiratory, and digestive system illnesses). However, the expected death rates from vascular disease, cirrhosis and other chronic liver diseases, and cancers are projected to be more in line with the SDG 40 × 30 target.

Mortality due to injuries has remained constant as a share of premature mortality over the past two decades. In 2010, non-road traffic and road traffic injuries, homicides, and suicides accounted for 53·1% of all deaths in individuals aged 5–19 years, and homicides were the leading cause of premature deaths for young adults (aged 20–49 years; appendix). However, owing to large variations in mortality rates across all age groups, homicide remains the most difficult cluster for which to predict trend behaviour over the next 15 years. Nevertheless, we find that homicides account for most of the projected shortfall in this cluster relative to the SDG 40 × 30 target. Projected deaths under the inertial scenario for road traffic accidents are in line with a 40% reduction in mortality, but will certainly fall short if we consider the SDG3 target for road safety, which is a 50% reduction in road traffic deaths and injuries by 2020.

**Figure 2:**
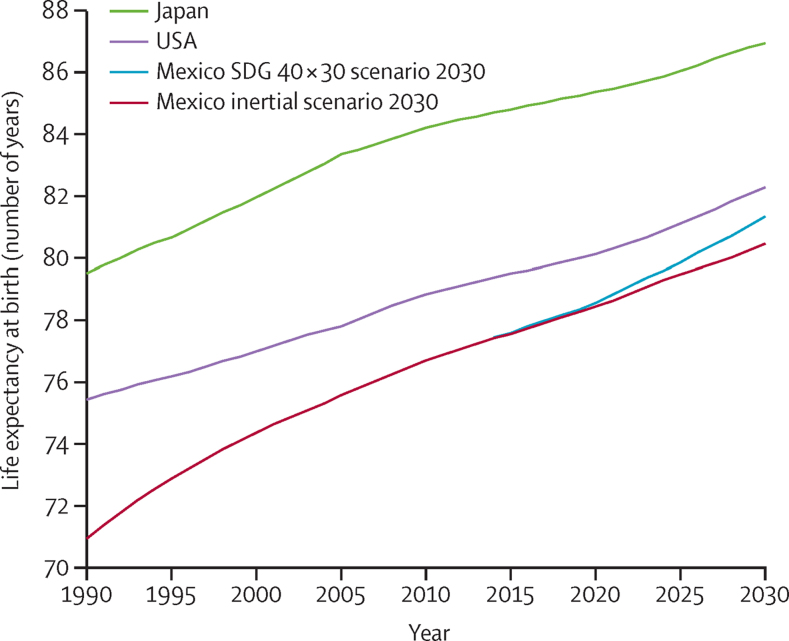
Mexico's potential to converge in life expectancy by 2030 Japan and USA estimates are based on UNPD data. Mexico's inertial forecast takes into account linear projections of trends in cause-specific mortality rates and United Nations population projections. Mexico's SDG 40 × 30 scenario considers an overall reduction of 40% in premature mortality (for individuals aged 0–69 years) by 2030. Own estimates using combined data from the United Nations, Department of Economic and Social Affairs, Population Division, World Population Prospects, 2015 Revision, and the National Institute of Statistics and Geography (INEGI), Statistics of mortality (1990–2014).

Mexico has the lowest life expectancy of all OECD countries and its present path is far from converging with better performing countries. From 2000 to 2013, the longevity gap between Mexico and the OECD average widened from 4 years to almost 6 years. Figure 2 shows past and projected trends for Japan (the best performer), the USA (underperformer), and Mexico, including the projected life expectancy under the inertial scenario (an additional 3·8 years) and the SDG 40 × 30 scenario (4·7 additional years), reaching 81·4 years by 2030.

In 2010, the difference in life expectancy between Mexico (76·7 years) and Japan (84·2 years) was 7·5 years. To close this gap in life expectancy with Japan, Mexico would need to substantially accelerate the annual rate of increase in life expectancy. This is an ambitious goal because the increase in life expectancy between 2000 and 2010 (2·3 years) was only two-thirds of that observed between 1990 and 2000 (3·5 years). A more reasonable goal for Mexico is to reach the United Nations estimate for life expectancy in the USA of 82·3 years by 2030.

**Figure 3:**
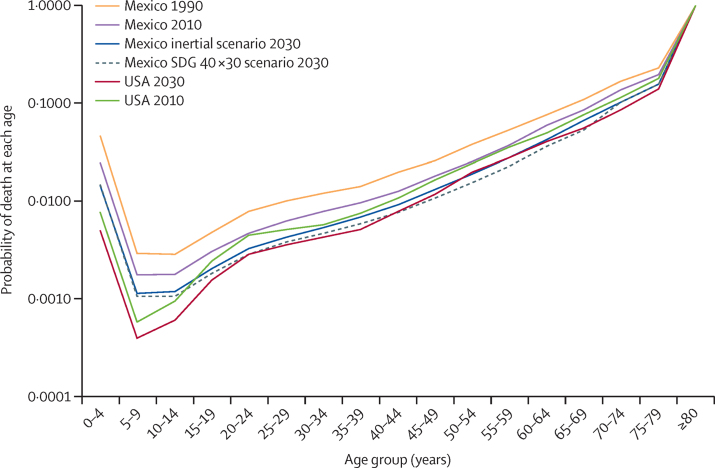
Mexico's potential to converge in mortality by age group, 1990, 2010, and 2030 Own estimates using data from: United Nations, Department of Economic and Social Affairs, Population Division, World Population Prospects, 2015 Revision.

By reviewing the age-specific gains in mortality projected under the SDG 40 × 30 scenario, we can assess the causes and trends in life expectancy in Mexico and identify the major shortfalls in mortality reduction that will need to be addressed for Mexico to converge with the USA. The differences in mortality rates by age group compared with the USA are presented in figure 3. The age-specific gains in mortality show that convergence can be achieved under the SDG 40 × 30 strategy if efforts are focused on the population aged 20–69 years. For older adults aged 50–69 years, mortality can even improve beyond the 2030 projection for the USA. Convergence for the young population would require substantial efforts that go beyond the SDG 40 × 30 target. The population aged older than 70 years is near convergence under UNPD-projected mortality, our assumption for this group for the SDG 40 × 30 scenario. However, given the high share of deaths in this group relative to total mortality, a greater reduction in mortality rates for this population group could result in a major shift in life expectancy. As expected, younger populations are still most affected by infectious diseases and childbirth mortality; homicides, non-road traffic injuries, and road traffic accidents affect mostly adults aged 20–49 years, and NCDs, such as diabetes, take their highest toll in older adults (aged 50 years and older).

## Discussion

To reduce premature mortality in Mexico by 40% by 2030 will require implementation of cost-effective health interventions, focusing on those age groups and health disorders in which inertial trends are projected to fall short of the SDG 40 × 30 target.

Maternal, neonatal, and child mortality gains are a success story and an important contributor to past increases in life expectancy, yet there is room to ensure that the expected favourable trends in this area are sustained to meet the SDG3. Mortality for these disorders is very concentrated in low-income and underserved populations who need to be reached with quality services (panel 1).

Present coverage rates for antenatal care (98·6%) and facility-delivery (94·5%) are high, but the quality of these services will need to be improved and standardised to address the wide variation in state practices and reduce mortality.^29^, ^39^, ^40^, ^41^ Efforts should focus on ensuring full adherence to WHO recommendations and clinical guidelines for antenatal and delivery care, including continuous care from early stages of pregnancy and compliance with caesarean best practices, especially focusing on the poorest areas of the country where deaths are highest.^42^, ^43^, ^44^

Improved access to neonatal care is also needed to reduce mortality in preterm babies. Focus should be placed on expanding adequate use of thermal care, continuous positive airway pressure therapy, surfactants for respiratory distress syndrome, oxygen therapy,^43^ breastfeeding, and skin-to-skin care.^45^, ^46^

To accelerate the reduction in mortality for the cluster of NCD deaths, we propose a three-pronged approach organised around the life cycle and stage of disease continuum, in line with WHO recommendations and universal health coverage goals.^47^

First, scaling up healthy lifestyle interventions that target underlying risk factors, such as economic incentives through taxation, is the most cost-effective strategy, although its full effect can only be seen in the long run. More rigorous fiscal and regulatory policies for tobacco, sugar, salt, and alcohol consumption should be introduced, building on existing policies and knowledge. These policies are powerful levers for reduction of NCDs, increase of revenue that can be allocated to the health sector, and can also be in favour of poor or low-income populations.^6^, ^48^, ^49^ In Mexico, the tobacco tax rate is still low compared with other countries and could be doubled to avert new smokers, especially young people.^50^, ^51^ In 2014, Mexico introduced a 10% tax on sugar-sweetened non-alcoholic beverages and an 8% tax on high-calorie processed foods. Although the tax was not earmarked to health and thus the budget for preventive care did not increase as a result, taxes increased prices and early evidence shows that consumption patterns are starting to change as a result.^52^, ^53^ Improving the availability and consumption of drinking water could help to decrease consumption of sugar-sweetened beverages. Finally, reducing sodium consumption, such as through regulating processed food, can be a cost-effective intervention to reduce cardiovascular mortality.^24^, ^47^, ^54^

A second set of NCD-related priorities are associated with more effective primary care-based prevention and early detection interventions combined with disease management to avoid further progression, ensure access to treatment and disease control, and ensure post-treatment follow-up to prevent recurrence. The mortality related to diabetes shows the present situation and the urgency of action. Within OECD countries, Mexico has one of the highest rates of hospital admissions due to uncontrolled diabetes, twice the OECD average.^17^ Poor access to, and poor performance of, primary care are to blame, including limited access to drugs and supplies and inadequate monitoring of clinical markers. Only 24% of patients with diabetes are considered to be under adequate control, and 49% of the diabetic population is unaware of their condition.^29^, ^55^ However, diabetes tends to be overused as the underlying cause of death for cardio-metabolic disease and its complications, thus the need for better classification of all causes of deaths is another pending challenge. Similarly, estimates suggest that more than 47% of adults with high blood pressure are unaware of their condition, and among adults aware of being hypertensive, only 74% were under treatment and only 51% had their blood pressure under control.^56^

A third set of priorities around NCDs that can deliver substantial short-term improvements in mortality are related to improved access to quality care for hospital-based treatment for patients, and timely emergency care. Even with great improvements in primary care, patients with vascular disorders or diabetes will continue to need access to hospital treatment and emergency care. There is insufficient access to hospital care, aggravated by the inefficient allocation of scarce hospital beds across patient needs.^16^, ^17^ Hospital admissions for congestive heart failure in adults are the lowest in OECD countries.^16^ Furthermore, uptake of effective procedures that are standard practice in other countries is comparatively low, such as coronary artery bypass grafting.^24^ Increased use of appropriate hospital-based interventions is therefore strongly recommended.

Chronic kidney disease prevention, including the control of associated risk factors, is feasible. In the short term, priority should be given to slow the progression towards end-stage renal failure in patients with chronic kidney disease. For more advanced disease stages, increased access to dialysis and kidney transplants is the only option. Both interventions are quite costly although transplants are more cost-effective, and evidence suggests that promoting transplants is desirable.^24^ Treatment for end-stage renal disease in Mexico is still restricted and insurance coverage for treatment varies. Increased access needs to be paired with standardised practice, especially across the different alternatives for dialysis treatment. Donor programmes also need to be scaled up to meet the demand for kidneys.

Finally, although projected mortality from cancer is in line with the SDG3 target, further reductions in premature mortality are both desirable and feasible. In particular, there are several cancers for which cost-effective interventions are available and that are highly preventable or curable when detected early. Access to treatment needs to be aligned with strengthened early detection and prevention at the primary care level to avoid heavily spending on very costly interventions that are likely to be ineffective due to the advanced stage of detection. In addition to interventions to reduce cancer risk factors, particularly smoking, priority interventions should include expanded coverage of human papilloma virus vaccination and the scale-up of screening, early detection, and treatment in early stages of cervical, breast, and colorectal cancer.^57^

Reduction of injury-related mortality will result in the greatest health gains in older children (aged 5–19 years) and young adults (aged 20–49 years). High mortality rates in children and young adults help to explain the present life expectancy difference between Mexico and other OECD countries, and are a contributing factor to the decreases in life expectancy gains observed between 2000 and 2010.

With increased motorisation associated with economic development, large efforts will be needed to meet the SDG3 target for road safety (a 50% reduction in road traffic deaths and injuries by 2020). Several effective policy options exist that can substantially reduce mortality, including setting and enforcement of speed limits, seatbelt requirements, drunk-driving laws, motorcyclists' helmet use and child safety restraint use, and improved vehicle safety standards and safer roads that account for the needs of all users (including pedestrians).^58^, ^59^ However, Mexico falls behind other countries in most of these measures.^35^, ^59^ Road safety legislation and enforcement is largely the responsibility of state and municipal authorities, and only a small number of states have legislation supporting these core preventive measures. Jalisco and Mexico City have much stronger legislation and enforcement and offer a positive example that could be used to design a national regulatory framework.^59^

Mexico experienced a large increase in homicide-related mortality rates during 2005–11 as a consequence of the national policy to fight drug traffic. The increase in fatalities explains the large reduction in life expectancy for men, especially in northern states such as Chihuahua, Sinaloa and Durango, and Guerrero and Nayarit, and a slowdown in the increase in life expectancy for women.^34^ Because many factors contribute to violence, tackling mortality from homicides requires an intersectoral approach. In terms of actions more directly amenable to health-sector leadership, and its coordination with other sectors, we propose that measures to reduce alcohol and drug consumption, as well as to promote healthy environments at the community level, are included in a broad strategy to reduce mortality from this cause.

To achieve convergence and meet the SDG3 targets, several systemic challenges will need to be addressed. The main challenge for Mexico's health system is how to organise the finance and delivery systems around these interventions in a way that ensures effective access and high quality, and standardised performance across the country (panel 2). In addition, addressing mortality due to the leading risk factors (high body-mass index, high fasting plasma glucose, alcohol and tobacco use, and high blood pressure) and injuries will require strong collaboration between the health sector and other sectors, such as education, labour, fiscal, and more.^68^

Although the aim of this study was to address shortfalls in premature mortality and thus stress timely policy actions needed to meet targets, important limitations should be noted. First and foremost, national averages will always hide important heterogeneity in population health needs. Our study disaggregated results by age group but not by sex, geography, ethnic group, or other socioeconomic variables known to be highly relevant to fully understand and assess policy options. Second, most policies will invariably be met with some degree of system-wide constraints, most notably financing and staffing restrictions. These might take years, if not decades, to overcome, and thus policy formulation might not easily move to the implementation phase in time to deliver substantial results by 2030. Finally, achieving gains in life expectancy will not guarantee healthier lifetimes free of disability and a more equitable distribution of health gains. Mental health problems as well as muscular-skeletal disabilities, many of which are highly prevalent in low-income populations, are not traditionally reflected in policies seeking to avert premature mortality. Finally, it is worth noting that the additional effort needed to avert deaths across age groups and diseases is different and changing over time as new cost-effective technologies are added to the health policy toolbox. Thus, the underlying assumption of reaching a 40% reduction across causes provides only a reference rate of decrease and not necessarily the most desirable or efficient pathways towards SDG3.

Mexico has achieved sustained progress in child, maternal, and infectious diseases-related mortality in the last three decades. However, it needs to adapt to the new health challenges in order to accelerate mortality reductions to attain SDG3. Urgent action is now needed to control NCDs and reduce fatal injuries, making a 40% reduction in premature mortality by 2030 feasible and putting Mexico back on a track of substantial life expectancy convergence with better performing countries.

## Figures and Tables

**Table 1:** Deaths and death rates by age group in 1990, 2000, 2010, and three scenarios for 2030

		**1990**		**2000**		**2010**		**2030**					
		Deaths	Rate*	Deaths	Rate*	Deaths	Rate*	Baseline deaths	Inertial scenario			SDG 40×30 target	
									Deaths†	Rate*	Change (%) *vs* baseline‡	Deaths§	Rate*
0–69 years		314 388	377·2	279 454	280·9	303 306	266·0	452 635	335 467	243·2	−25·9	271 581	196·9
	0–4 years	108 934	941·7	72 500	593·3	57 603	494·3	51 939	30 124	286·7	−42·0	31 163	296·6
	5–19 years	22 145	68·7	16 241	48·2	15 170	42·9	14 424	9642	28·6	−33·2	8654	25·7
	20–49 years	82 538	258·9	82 792	190·0	94 348	180·1	116 925	87 324	134·5	−25·3	70 155	108·1
	50–69 years	100 771	1315·4	107 921	1079·7	136 185	933·2	269 347	208 377	722·0	−22·6	161 608	559·9
70 years and over		149 190	6567·2	199 308	5965·6	252 149	5496·8	559 556	476 063	4676·6	−14·9	476 063	4676·6
Total		463 578	541·5	478 762	465·7	555 455	468·3	1 012 191	811 530	547·8	−19·8	747 644	504·7

Own estimates using data from the United Nations, Department of Economic and Social Affairs, Population Division, World Population Prospects, 2015 Revision.

* All death rates are expressed per 100 000 population.

† United Nations Population Division (UNPD) death estimates for 2030.

‡ Percentage reduction of deaths under the estimated inertial scenario based on UNPD death estimates, relative to the baseline scenario.

§ Death rates for 2030 population assuming a flat 40% reduction in the baseline number of deaths for the 0–69 years age group and assuming the inertial rate for the 70 years and older age group.

**Table 2:** Premature deaths and death rates by cause in 1990, 2000, 2010, and three scenarios for 2030

				**1990**		**2000**		**2010**		**2030**					
				Deaths	Rate*	Deaths	Rate*	Deaths	Rate*	Baseline deaths	Inertial scenario			SDG 40×30 target	
											Deaths†	Rate*	Change *vs* baseline (%)‡	Deaths§	Rate*
Ages 0–69 years				314 388	377·2	279 454	280·9	303 306	266·0	452 635	335 467	243·2	−25·9%	271 581	196·9
	Communicable, perinatal, maternal, or nutritional causes			108 864	130·6	66 176	66·5	51 637	45·3	57 892	27 253	19·8	−52·9%	34 735	25·2
		Newborn and child health (ages 0–4 years)		81 433	704·0	47 119	385·6	33 255	285·4	29 985	11 386	108·4	−62·0%	17 991	171·2
			Asphyxia and birth trauma	19 572	169·2	19 028	155·7	13 574	116·5	12 239	3285	31·3	−73·2%	7343	69·9
			Acute respiratory infections	16 866	145·8	6833	55·9	3949	33·9	3561	0	0·0	−100·0%	2137	20·3
			Intestinal infections	18 308	158·3	4029	33·0	1450	12·4	1307	0	0·0	−100·0%	784	7·5
			Perinatal and other nutritional and communicable causes	26 687	230·7	17 229	141·0	14 282	122·6	12 878	8101	77·1	−37·1%	7727	73·5
		Maternal and other nutritional and communicable causes (ages 5–69 years)		27 431	38·2	19 057	21·8	18 382	18·0	27 907	15 867	12·5	−43·1%	16 744	13·1
	Non-communicable diseases			147 562	177·1	163 493	164·4	191 589	168·0	316 871	244 198	177·0	−22·9%	190 123	137·8
		Vascular, diabetes, and related disorders		72 714	87·3	84 816	85·3	103 574	90·8	183 561	137 831	99·9	−24·9%	110 137	79·8
			Vascular	35 304	42·4	34 456	34·6	41 145	36·1	72 349	52 950	38·4	−26·8%	43 409	31·5
			Diabetes	16 142	19·4	25 235	25·4	38 109	33·4	70 435	69 188	50·2	−1·8%	42 261	30·6
			Cirrhosis and other chronic liver diseases	16 191	19·4	19 981	20·1	18 350	16·1	31 250	7729	5·6	−75·3%	18 750	13·6
			Renal	5077	6·1	5144	5·2	5970	5·2	9527	7964	5·8	−16·4%	5716	4·1
		Cancers		28 640	34·4	34 138	34·3	37 669	33·0	63 471	42 847	31·1	−32·5%	38 083	27·6
		Other non-communicable diseases		46 208	55·4	44 539	44·8	50 346	44·2	69 839	63 521	46·0	−9·0%	41 903	30·4
	Injuries			57 962	69·6	49 785	50·1	60 080	52·7	77 872	64 016	46·4	−17·8%	46 723	33·9
		Homicides		14 972	18·0	10 709	10·8	22 618	19·8	28 946	37 828	27·4	30·7%	17 368	12·6
		Non-road traffic injuries		27 138	32·6	22 114	22·2	19 265	16·9	25 208	9317	6·8	−63·0%	15 125	11·0
		Road traffic injuries		13 918	16·7	13 565	13·6	13 977	12·3	18 271	10 530	7·6	−42·4%	10 963	7·9
		Suicides		1934	2·3	3397	3·4	4220	3·7	5447	6341	4·6	16·4%	3268	2·4
Ages 70 years and over				149 190	6567·2	199 308	5965·6	252 149	5496·8	559 556	476 063	4676·6	−14·9%	476 063	4676·6
Total				463 578	541·5	478 762	465·7	555 455	468·3	1 012 191	811 530	547·8	−19·8%	747 644	504·7

Own estimates using combined data from the United Nations, Department of Economic and Social Affairs, Population Division, World Population Prospects, 2015 Revision, and the National Institute of Statistics and Geography (INEGI), Statistics of mortality (1990–2014).

* All death rates are expressed per 100 000 population.

† The estimated and adjusted number of deaths for 2030 use United Nations Population Division (UNPD) population projections and implied death rates for 2030 with linear regressions based on cause-specific and age-specific mortality rates for years 2000–14.

‡ Percentage reduction of deaths under the estimated inertial scenario based on UNPD death estimates, relative to the baseline scenario.

§ Death rates for 2030 population assuming a flat 40% reduction in the baseline number of deaths across age groups. The list of the International Classification of Diseases, 9th Revision and the International Classification of Diseases, 10th Revision codes used to group deaths by major cause is included in the appendix.

## Panel 1 Mexico's baseline for SDG3: progress in health status and future challenges9–38

Over the past 30 years, Mexico has achieved major improvements in maternal and child health, and gains in life expectancy. This is the result of a combination of factors, including strong health leadership, an emphasis on evidence-based policy implementation, and substantial and well targeted investment in cost-effective health interventions. These interventions range from oral rehydration therapy, universal vaccination, clean water, and vector control to HIV/AIDS prevention and conditional cash transfers to promote regular clinic visits among the poorest population. The combined effect of these measures helped to reduce maternal mortality from 88·7 deaths per 100 000 livebirths in 1990 to 38·9 per 100 000 livebirths in 2014, reaching 75% of the Millennium Development Goal (MDG) target for 2015. Under-5 child mortality fell 63·2% from 41·0 per 1000 livebirths in 1990 to 15·1 in 2014, close to the corresponding MDG target of 13·7.

Despite these substantial gains, Mexico is behind OECD countries in several key areas. First, maternal, newborn, and child health (MNCH) performance is comparatively poor because several child health disorders and preventable diseases still account for an unacceptable share of total mortality. Neonatal mortality in Mexico is more than twice the rate of that in the USA (8·2 and 3·6 deaths per 1000 livebirths, respectively), the infant mortality rate is three times higher than the OECD average (13 and 3·8 deaths per 1000 livebirths in 2013, respectively), and the under-5 mortality rate is 16·8 compared with 6·6 in the USA. Poor MNCH performance is to a large extent a result of inequalities in income and other social determinants of health, as well as an absence of access to quality health services. Substantial inequalities in health across states persist, with poorer, southern states such as Oaxaca, Chiapas, and Guerrero falling behind in maternal and child health outcomes as well as in other indicators of health status, such as life expectancy.

Second, Mexico has a high and rising incidence of many non-communicable disease (NCDs) as a result of its aging population and an increased prevalence of related risk factors. NCDs now account for more than three-quarters of total mortality. Diabetes and cardiovascular disease alone account for 33·4% of adult mortality. The diabetes prevalence rate in adults aged older than 20 years is the highest in OECD countries, and twice the OECD average. The mortality rate from ischaemic heart disease is 1·2 times the OECD average. Although the rates of the most important related risk factors, overweight and obesity, are stabilising in many high-income countries, they continue to grow in Mexico. Of all OECD countries, Mexico, along with the USA, has the highest rates of overweight and obesity; prevalence in adults stood at more than 70% in 2012. Cancers are also emerging as a more frequent cause of death. In particular, breast cancer is the second leading cause of death (after diabetes) of women aged 30–54 years. Similar to MNCH, regional inequalities in NCD rates are also substantial, although the geographical pattern of inequality is different. The northern and central regions and urban areas of Mexico have the highest rates of obesity and diabetes, and mortality from ischaemic heart disease. Death rates from NCDs tend to be higher, however, in low-income populations living in Mexico and incidence and mortality from cervical cancer is concentrated in the poorest states.

Third, Mexico has experienced a large increase in deaths due to injuries, particularly those that are violence-related. Although violent deaths have begun to decrease, they remain higher than in previous years. Violence-related mortality is highly concentrated in a few regions. The high rates of homicide in states like Durango, Chihuahua, and Guerrero are a major cause of the decreasing life expectancy for young men. Mortality due to accidents, particularly road traffic accidents, is also a substantial cause of preventable mortality.

As a result, the increase in life expectancy has slowed. Although Mexico gained on average 4·2 months per year in life expectancy between 1990 and 2000, from 2000 to 2015 it gained only 2 months per year, and is far from converging with OECD countries. In 2015, life expectancy in Mexico reached 77 years, whereas in Japan (the best performer among OECD countries) it was 83·7 years and in the USA (a not-so-good performer) it was 79·2 years.

## Panel 2 Systemic challenges and opportunities to meet SDG360–67

Despite great efforts to increase public health spending, Mexico still spends less on health, as a proportion of gross domestic product (GDP), than other OECD countries. In 2013, Mexico's total health expenditure was 6·2% of GDP, compared with the 8·9% OECD average. The public component of health expenditures is also substantially lower in Mexico, at 3·2% of GDP, compared with 6·5% in OECD countries, and out-of-pocket spending on health remains relatively high, making up 44·7% of total health expenditures in 2013 compared with 20% in OECD countries. Despite the expansion of health coverage through Seguro Popular (a public health insurance in Mexico) in the past decade, new efforts are neeeded not only to optimise returns on health investments but also to further improve the provision of financial risk protection.

At the same time, the country faces challenges in cost-containment, not least because high rates of chronic illness come at substantial economic cost. Costs associated with the primary obesity-related diseases are projected to increase from US$880 million in 2013 to $1·2 billion by 2030. The costs (direct and indirect) associated with diabetes and its complications account for 2·25% of Mexico's GDP. And in 2008, the annual cost of treatment for four obesity-related diseases (colorectal malignant tumours, malignant breast tumours, diabetes, and cardiovascular disease) is estimated to amount to a third of the country's public health-care budget. Unless effective glycaemic control among patients with diabetes becomes commonplace, fatal diabetic complications will continue being not only a health burden, but also an economic one.

In these circumstances, to meet the SDG3 targets, Mexico will need to increase its health investments. In line with international targets, this would mean reaching a level for government funding of 5% of GDP. But Mexico will also need to improve efficiency, which requires financial and institutional reform and political will to ensure that resources are allocated in an optimum way. Optimum allocation will require changes in how resources are distributed across different parts of the country, as well as how they are allocated across institutions, interventions, and health-care providers. Attention should be focused on poor-performing states and on the emerging health crisis of NCDs and injuries. A so-called whole health systems universal health coverage approach that includes all health system functions and major actors, not limited to Seguro Popular or health insurance for financial protection alone, will also be needed.

Dealing with NCDs and injuries presents substantial operational and financial challenges for the health system, as well as opportunities to achieve SDG3 following a 40×30 strategy. Mexico will need to expand health insurance packages beyond WHO's suggested best-buy interventions for NCDs to include a much wider range of early detection, treatment, and management of these diseases. Strengthening primary care would be the best route for the system to maintain a focus on both maternal and child disorders while dealing with NCDs and their associated risk factors. The effective treatment of NCDs will require restructuring service delivery in a way that enables not only a shift of attention towards prevention, but also a dramatic move from episodic care to continuous care for chronic disorders, including survivorship and palliative care.

Mexico's path to SDG 40×30 should be anchored in both deepening entitlements to a broader package of care services with financial protection while at the same time extending the gains achieved in MNCH with core efforts in primary care. This holistic approach echoes the SDGs in maximising the synergies between health gains and poverty reduction.
