# Supplementary material for: Mexico's path towards the Sustainable Development Goal for health: an assessment of the feasibility of reducing premature mortality by 40% by 2030
Source: Lancet Glob Health. 2016 Aug 30;4(10):e714–25. doi: 10.1016/S2214-109X(16)30181-4 (PMC5024342; doi:10.1016/S2214-109X(16)30181-4)
Supplement: Supplementary appendix [file mmc1.pdf]

THE LANCET Global Health

Supplementary appendix

This appendix formed part of the original submission and has been peer reviewed. We post it as supplied by the authors.

Supplement to: González-Pier E, Barraza-Lloréns M, Beyeler N, et al. Mexico's path towards the Sustainable Development Goal for health: an assessment of the feasibility of reducing premature mortality by 40% by 2030. *Lancet Glob Health* 2016; published online Aug 30. http://dx.doi.org/10.1016/S2214-109X(16)30181-4.

Appendix to Mexico's path towards the Sustainable Development Goal for health: an assessment of the feasibility of reducing premature mortality by 40% by 2030. Lancet Glob Health 2016 http://dx.doi.org/10.1016/S2214-109X(16)30181-4

**SDG 3: Ensure healthy lives and promote well-being for all at all ages (2015)**	**The Lancet Commission on Investing in Health (CIH) (2013)**	**Norheim and colleagues (2014)**
**2030 targets and means of implementation ***	**2035 targets ** (interim 2030 targets)**	**2030 targets**
**With a quantitative and/or specific quantifiable measure**		**Overarching target for mortality: avoid 40% of the premature deaths in each country (the deaths before age 70 years that would occur in that country's 2030 population at its 2010 death rates)**
By 2030, reduce the global maternal mortality ratio to less than 70 per 100,000 live births.	83 deaths per 100,000 live births (interim target of 94 by 2030).	Avoid two-thirds of child and maternal deaths.
By 2030, end preventable deaths of newborns and children under 5 years of age, with all countries aiming to reduce neonatal mortality to at least as low as 12 per 1,000 live births and under-5 mortality to at least as low as 25 per 1,000 live births.	16 deaths in under- 5 per 1,000 live births (interim target of 20 by 2030).	
By 2030, end the epidemics of AIDS, tuberculosis, malaria and neglected tropical diseases and combat hepatitis, water-borne diseases and other communicable diseases.	Tuberculosis: 4 per 100,000 population per year. HIV/AIDS: 8 per 100,000 population per year.	Avoid two-thirds of HIV, tuberculosis, and malaria deaths.
By 2020, halve the number of global deaths and injuries from road traffic accidents.		Avoid a third of premature deaths from other communicable diseases or injuries.
By 2030, reduce by one-third premature mortality from non-communicable diseases through prevention and treatment and promote mental health and wellbeing.		Avoid a third of the premature deaths from NCDs.
By 2030, substantially reduce the number of deaths and illnesses from hazardous chemicals and air, water and soil pollution and contamination.		
**Instrumental, qualitative or without a quantifiable measure**		**Overarching target for health care at all ages**
By 2030, ensure universal access to sexual and reproductive health-care services, including for family planning, information and education, and the integration of reproductive health into national strategies and programmes.		Extend access to cost-effective, affordable treatment, including mental health and sexual, reproductive and perinatal services (with targets that are appropriately country-specific).
Achieve universal health coverage, including financial risk protection, access to quality essential health-care services and access to safe, effective, quality and affordable essential medicines and vaccines for all.		
Strengthen the prevention and treatment of substance abuse, including narcotic drug abuse and harmful use of alcohol.		
Strengthen the implementation of the World Health Organization Framework Convention on Tobacco Control in all countries, as appropriate.		
Support the research and development of vaccines and medicines for the communicable and non-communicable diseases that primarily affect developing countries, provide access to affordable essential medicines and vaccines, in accordance with the Doha Declaration on the TRIPS Agreement and Public Health, which affirms the right of developing countries to use to the full the provisions in the Agreement on Trade Related Aspects of Intellectual Property Rights regarding flexibilities to protect public health, and, in particular, provide access to medicines for all.		
Substantially increase health financing and the recruitment, development, training and retention of the health workforce in developing countries, especially in least developed countries and small island developing States.		
Strengthen the capacity of all countries, in particular developing countries, for early warning, risk reduction and management of national and global health risk.		

* The listing of SDG 3 targets and means of implementation does not correspond to the original listing by the United Nations. They have been classified based on whether they are defined on a quantifiable/measurable basis. Some targets and means of implementation are qualitative, such as strengthening the prevention and treatment of substance abuse, while others are too comprehensive, vague and/or unattainable. For example, aiming to "end by 2030 the epidemics of AIDS, tuberculosis, malaria and neglected tropical diseases and combat hepatitis, water-borne diseases and other communicable diseases" is highly desirable but it could prove far from feasible. Even if adequate efforts –including financial and political–, are put in place, the breadth of this target and the challenges intrinsic to service delivery could make it beyond reach. ** The CIH targets represent an average across low-income and lower-middle-income countries. Own elaboration based on: United Nations. Sustainable Development Goals. 17 goals to transform our world. http://www.un.org/sustainabledevelopment/summit/ (accessed Nov 27, 2015). Jamison DT, Summers LH, Alleyne G, et al. Global health 2035: a world converging within a generation. Lancet 2013; 382: 1898–955. Norheim OF, Jha P, Admasu K, et al. Avoiding 40% of the premature deaths in each country, 2010–30: review of national mortality trends to help quantify the UN sustainable development goal for health. Lancet 2015; 385: 239–52.

***Table A.1*: United Nations Sustainable Development Goal for health (SDG 3) and other post-2015 global health proposals**

Cause	ICD-9	ICD-10
**Communicable, perinatal, maternal, or nutritional causes**	**001-139, 243, 260- 269,279.5, 279.6, 280-285,320-326, 381-382,460-466, 480-487, 614-616, 630-676, 760-779**	**A00-B99, G00-G09, N70-N76, J00-J06, J10-J18, J20-J22, H65-H69, O00-O99, P00-P96, E00, E40-E64, D50-D64**
Newborn and child health (ages 0 -4 years)	760-763, 766-770, 771.0-771.2, 771.4-779, 001-009, 460-466, 480-487	P00-P04, P08, P10-P15, P20-P29, P35-P96, A00-A09, J00-J06, J10-J18, J20-J22
Asphyxia and birth trauma	763, 767-770	P03, P10-P15, P20-P28
Acute respiratory infections	460-466, 480-487	J00-J06, J10-J18, J20-J22
Intestinal infections	001-009	A00-A09
Perinatal and other nutritional and communicable causes	260-269, 760-762, 766, 771.0-771.2, 771.4-779	E00, E40-E64, D50-D64, P00-P02, P04, P08, P29, P35-P96
Maternal and other nutritional and communicable causes (ages 5-69 years)	630-676, 010-139, 243, 260- 269, 279.5, 279.6, 280-285,320-326, 381-382, 614-616, 763-765, 771-779, except 771.0, 771.2, 771.4	A10-B99, E00, E40-E64, D50-D64, G00-G09, N70-N76, H65-H69, O00-O99, P05-P09, P16-P19, P30-P34, E00, E40-E64, D50-D64
**Non-communicable diseases**	**140-242, 244-259, 270-279.4, 279.8-279.9, 286-319, 330-380, 383-459, 470-478, 490-613, 617-629, 680-759**	**C00-C97, D00-D48, D65-D89, E01-E07, E10-E16, E20-E34, E65-E89, F01-F99, G06-G99, H00-H61, H70-H95, I00-I99, J30-J99, K00-K92, N00-N64, N80-N99, L00-L99, M00-M99, Q00-Q99**
Vascular, diabetes and related disorders	250, 390-459, 571, 580-589	E10-E14, I00-I99, K70, K72.1, K73, K74, K76, N00-N19, C00-D48
Vascular	390-459	I00-I99
Diabetes	250	E10-E14
Cirrhosis and other chronic liver diseases	571	K70, K72.1, K73, K74, K76
Renal	580-589	N00-N19
Cancers	140-208, 210-239	C00-D48
Other non-communicable diseases	140-242, 244-259, 270-279.4, 279.8-279.9, 286-319, 330-380, 383-459, 470-478, 490-613, 617-629, 680-759	D65-D89, E01-E07, E15-E16, E20-E34, E65-E89, F01-F99, G06-G99, H00-H61, H70-H95, J30-J99, K00-K69, K71, K75, K77-K92, N20-N64, N80-N99, L00-L99, M00-M99, Q00-Q99
**Injuries**	**E800-E978, E990-E999**	**V01-Y09, Y35-Y89**
Homicides	E960-E969	X85-Y09, Y87.1
Non-road traffic injuries	E800-E809, E820-E949, E970-E978, E990-E999	V01-Y09, Y35-Y89, except (V02-V04 (.1, .9), V09.2-V09.3, V09.9, V12-V14 (.3-.9), V19.4-V19.6, V20-V28 (.3-.9), V29-V79 (.4-.9), V80.3-V80.5, V81.1, V82.1, V83-V86 (.0-.3), V87.0-V87.8, V89.2, V89.9 , Y85.0, X85-Y09, Y87.1, X60-X84, Y87.0)
Road traffic injuries	E810-E819	V02-V04 (.1, .9), V09.2-V09.3, V09.9, V12-V14 (.3-.9), V19.4-V19.6, V20-V28 (.3-.9), V29-V79 (.4-.9), V80.3-V80.5, V81.1, V82.1, V83-V86 (.0-.3), V87.0-V87.8, V89.2, V89.9, Y85.0
Suicides	E950-E959	X60-X84, Y87.0

***Table A.2*: List of ICD codes used to group causes of death into 15 major categories**

Appendix to Mexico’s path towards the Sustainable Development Goal for health: an assessment of the feasibility of reducing premature mortality by 40% by 2030. Lancet Glob Health 2016 http://dx.doi.org/10.1016/S2214-109X(16)30181-4

Age group / Cause	1990		2000		2010		2030					
	Deaths	Rate*	Deaths	Rate*	Deaths	Rate*	Baseline deaths	Inertial scenario			SDG 40x30 target	
								Deaths †	Rate*	Change *vs* baseline (%) ‡	Deaths ¶	Rate*
**Ages 0-69 years**	**314,388**	**377.2**	**279,454**	**280.9**	**303,306**	**266.0**	**452,635**	**335,467**	**243.2**	**-25.9**	**271,581**	**196.9**
Ages 0-4 years	***108,934***	***941.7***	***72,500***	***593.3***	***57,603***	***494.3***	***51,939***	***30,124***	***286.7***	***-42.0***	***31,163***	***296.6***
Asphyxia and birth trauma	19,572	169.2	19,028	155.7	13,574	116.5	12,239	3,285	31.3	-73.2	7,343	69.9
Acute respiratory infections	16,866	145.8	6,833	55.9	3,949	33.9	3,561	0	0	-100.0	2,137	20.3
Non road traffic injuries	4,280	37.0	4,524	37.0	2,781	23.9	2,508	837	8.0	-66.6	1,505	14.3
Intestinal infections	18,308	158.3	4,029	33.0	1,450	12.4	1,307	0	0	-100.0	784	7.5
Vascular	1,551	13.4	765	6.3	1,244	10.7	1,122	1,434	13.6	27.8	673	6.4
Other causes	*48,357*	418	*37,321*	*305.4*	*34,605*	*296.9*	*31,202*	*24,568*	*233.8*	*-21.3*	*18,721*	*178.2*
Ages 5-19 years	***22,145***	***68.7***	***16,241***	***48.2***	***15,170***	***42.9***	***14,424***	***9,642***	***28.6***	***-33.2***	***8,654***	***25.7***
Non road traffic injuries	5,178	16.1	3,489	10.4	2,571	7.3	2,445	243	0.7	-90.1	1,467	4.4
Homicides	1,998	6.2	1,437	4.3	2,377	6.7	2,260	3,058	9.1	35.3	1,356	4.0
Road traffic injuries	2,858	8.9	2,617	7.8	2,296	6.5	2,183	921	2.7	-57.8	1,310	3.9
Cancers	1,732	5.4	2,155	6.4	1,874	5.3	1,782	1,136	3.4	-36.3	1,069	3.2
Suicides	342	1.1	665	2.0	815	2.3	775	1,071	3.2	38.2	465	1.4
Other causes	*10,037*	*31.1*	*5,878*	*17.5*	*5,237*	*14.8*	*4,979*	*3,214*	*9.5*	*-35.5*	*2,987*	*8.9*
Ages 20-49 years	***82,538***	***258.9***	***82,792***	***190.0***	***94,348***	***180.1***	***116,925***	***87,324***	***134.5***	***-25.3***	***70,155***	***108.1***
Homicides	10,333	32.4	7,557	17.3	17,605	33.6	21,818	28,490	43.9	30.6	13,091	20.2
Cancers	8,247	25.9	10,459	24.0	10,760	20.5	13,335	8,539	13.2	-36.0	8,001	12.3
Non road traffic injuries	13,025	40.9	10,302	23.6	9,833	18.8	12,186	4,803	7.4	-60.6	7,312	11.3
Vascular	8,543	26.8	8,163	18.7	9,457	18.1	11,720	9,758	15.0	-16.7	7,032	10.8
Road traffic injuries	7,780	24.4	7,888	18.1	8,120	15.5	10,063	6,301	9.7	-37.4	6,038	9.3
Other causes	*34,610*	*108.6*	*38,423*	*88.2*	*38,573*	*73.6*	*47,803*	*29,433*	*45.3*	*-38.4*	*28,682*	*44.2*
Ages 50-69 years	***100,771***	***1,315.4***	***107,921***	***1,079.7***	***136,185***	***933.2***	***269,347***	***208,377***	***722.0***	***-22.6***	***161,608***	***559.9***
Diabetes	13,231	172.7	20,429	204.4	31,466	215.6	62,234	61,020	211.4	-2.0	37,340	129.4
Vascular	24,281	316.9	24,932	249.4	29,758	203.9	58,855	41,073	142.3	-30.2	35,313	122.4
Cancers	17,768	231.9	20,406	204.1	23,957	164.2	47,382	32,363	112.1	-31.7	28,429	98.5
Cirrhosis	8,887	116.0	10,998	110.0	11,580	79.4	22,903	7,457	25.8	-67.4	13,742	47.6
Non road traffic injuries	4,655	60.8	3,799	38.0	4,080	28.0	8,069	3,435	11.9	-57.4	4,841	16.8
Other causes	*31,949*	*417.0*	*27,357*	*273.7*	*35,344*	*242.2*	*69,904*	*63,029*	*218.4*	*-9.8*	*41,942*	*145.3*
**Ages 70 years and over**	**149,190**	**6,567.2**	**199,308**	**5,965.6**	**252,149**	**5,496.8**	**559,556**	**476,063**	**4,676.6**	**-14.9**	**476,063**	**4,676.6**
**Total**	**463,578**	**541.5**	**478,762**	**465.7**	**555,455**	**468.3**	**1,012,191**	**811,530**	**547.8**	**-19.8**	**747,644**	**504.7**

Own estimates using combined data from the United Nations, Department of Economic and Social Affairs, Population Division, World Population Prospects, 2015 Revision and the National Institute of Statistics and Geography (INEGI), Statistics of mortality (1990–2014). * All death rates are expressed per 100,000 population. † The estimated and adjusted number of deaths for 2030 use United Nations Population Division (UNPD) population projections and implied death rates for 2030 with linear regressions based on cause-specific and age-specific mortality rates for years 2000–14. ‡ Percentage reduction of deaths under the estimated inertial scenario based on UNPD death estimates, relative to the baseline scenario. ¶ Death rates for 2030 population assuming a flat 40% reduction in the baseline number of deaths across age groups (0-69 years), and assuming the inertial mortality rate for the group 70 years and over. The list of the International Classification of Diseases, 9th Revision and the International Classification of Diseases, 10th Revision codes used to group deaths by major cause is also included in the appendix.

Table A.3: **Premature deaths (0-69 years) and death rates by age group and five leading causes of death in 1990, 2000, 2010, and three scenarios for 2030**

Appendix to Mexico's path towards the Sustainable Development Goal for health: an assessment of the feasibility of reducing premature mortality by 40% by 2030. Lancet Glob Health 2016 http://dx.doi.org/10.1016/S2214-109X(16)30181-4
